# Supplementary material for: A retrospective study: cardiac MRI of fulminant myocarditis in children—can we evaluate the short-term outcomes?
Source: PeerJ. 2016 Dec 15;4:e2750. doi: 10.7717/peerj.2750 (PMC5162402; doi:10.7717/peerj.2750)
Supplement: Table S1 — “-”, negative results; “+”, positive result. EBV, Epstein-Barr virus; CMV, Cytomegalovirus; HSV, Herpes simplex virus; RUB, Rubella; PIV, Parainfluenza virus; RSV, Respiratory syncytial virus–FF1B; EBNA, Epetein-Bair virus nuclear antigen; EBEA, Epetein-Bair virus early antigen; TOX, Toxoplasma. [file peerj-04-2750-s002.docx]

Table S1 The viral serology of eight FM children.

|  | A | B | C | D | E | G | H | I |
| --- | --- | --- | --- | --- | --- | --- | --- | --- |
| **DNA/RNA** | | | | | | | | |
| EBV DNA | - | - |  | - | - | - | - | - |
| CMV DNA | - | - |  | - | - | - |  | - |
| HSV DNA | - | - |  |  |  |  |  |  |
| Adenovirus DNA |  | - |  |  |  |  |  |  |
| Parvo B19 |  | - |  | - |  |  | - | - |
| RUB RNA | - |  |  |  |  |  |  |  |
| Coxsackie RNA |  | - |  |  |  |  |  |  |
| **IGM/IGG** | | | | | | | | |
| Adenovirus IGM | - | - | - |  | - | - |  |  |
| RSV IGM | - | - | - |  | - | - |  |  |
| Influenza A and B IGM | - | - | - |  | - | - |  |  |
| PIV IGM | - | - | - |  | - | - |  |  |
| Parvo B19 IGM |  |  | - |  |  | - |  |  |
| Coxsackie IGM |  |  | - |  | - | - | - | - |
| Varicella -IGG |  | + | + | + | + | + |  | + |
| Varicella -IGM |  | - | - | - | - | - |  | - |
| EBNA-IGG |  | - | - | - | - | - |  | - |
| EBEA-IGG |  | + | + | + | + | + |  | + |
| TOXIGM |  | - |  | - |  | - | - | - |
| TOXIGG |  | - |  | - |  | - | - | - |
| RUBIGM |  | - |  | - |  | - | - | - |
| RUBIGG |  | + |  | + |  | + | + | + |
| CMVIGM |  | - | - | - |  | - | - | - |
| CMVIGG |  | + | - | + |  | + | + | + |
| HSV-IGM |  | - | - | - |  | - | - | - |
| HSV-IGG |  | - | + | - |  | + | + | + |

“-”, negative results; “+”, positive results

EBV, Epstein-Barr virus; CMV, Cytomegalovirus; HSV, Herpes simplex virus; RUB, Rubella; PIV, Parainfluenza virus; RSV, Respiratory syncytial virus； EBNA, Epetein-Bair virus nuclear antigen; EBEA, Epetein-Bair virus early antigen; TOX, Toxoplasma.
