# Supplementary figures and images for: A retrospective study: cardiac MRI of fulminant myocarditis in children—can we evaluate the short-term outcomes?
Source: PeerJ. 2016 Dec 15;4:e2750. doi: 10.7717/peerj.2750 (PMC5162402; doi:10.7717/peerj.2750)

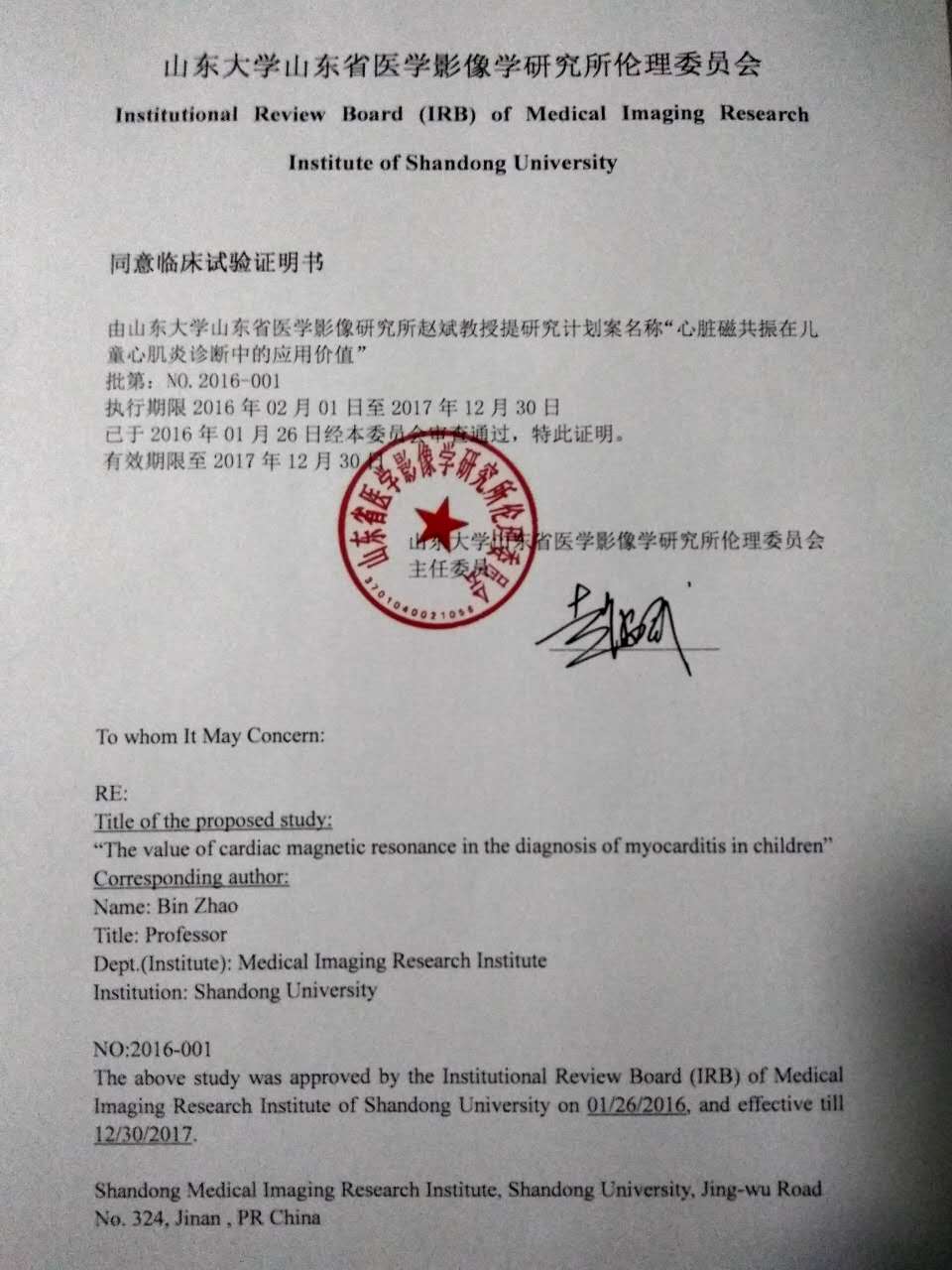

Supplement: Supplemental Information 3 [file peerj-04-2750-s003.jpg]
